# 1-{(*Z*)-1-[3-(4-Bromo­phen­oxy)prop­oxy]-1-(2,4-difluoro­phen­yl)prop-1-en-2-yl}-1*H*-1,2,4-triazol-4-ium nitrate

**DOI:** 10.1107/S1600536812024154

**Published:** 2012-06-13

**Authors:** Fei Shen, Song Guo, Yuan-yuan Luan, Kai Wang, Yong-hong Hu

**Affiliations:** a Jiangsu Engineering Technology Research Center of Polypeptide Pharmaceutical, College of Life Science and Pharmaceutical Engineering, Nanjing University of Technology, Xinmofan Road No. 5 Nanjing, Nanjing 210009, People’s Republic of China; b College of Pharmaceutical Science, Nanjing University of Technology, Xinmofan Road No. 5 Nanjing, Nanjing 210009, People’s Republic of China

## Abstract

In the title mol­ecular salt, C_20_H_19_BrF_2_N_3_O_2_^+^·NO_3_^−^, the N atom at position 4 of the heterocyclic ring is protonated. The triazole ring makes dihedral angles of 96.6 (4) and 54.4 (3)° with the 4-bromo­phenyl and 2,4-difluoro­phenyl rings, respectively, and the mol­ecule adopts a *Z* conformation about the C=C double bond. In the crystal, cations and anions are linked by N—H⋯O and C—H⋯O hydrogen bonds.

## Related literature

For background to the uses of triazole derivatives, see: Jeu *et al.* (2003 ▶); Fromtling & Castaner (1996 ▶). For further synthetic details, see: Ludwig & Kurt (1985 ▶). 
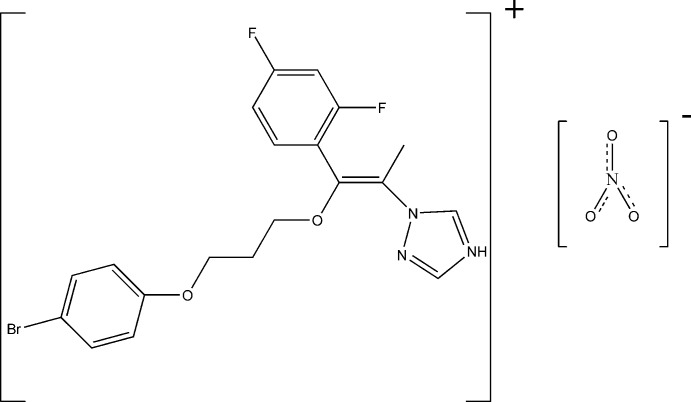


## Experimental

### 

#### Crystal data

C_20_H_19_BrF_2_N_3_O_2_^+^·NO_3_^−^
*M* *_r_* = 513.30
Triclinic, 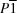

*a* = 8.3030 (17) Å
*b* = 8.4260 (17) Å
*c* = 16.170 (3) Å
α = 91.10 (3)°
β = 95.80 (3)°
γ = 102.30 (3)°
*V* = 1098.7 (4) Å^3^
*Z* = 2
Mo *K*α radiation
μ = 1.93 mm^−1^
*T* = 293 K
0.20 × 0.10 × 0.10 mm

#### Data collection

Enraf–Nonius CAD-4 diffractometer
Absorption correction: ψ scan (North *et al.*, 1968 ▶) *T*_min_ = 0.699, *T*_max_ = 0.831
4329 measured reflections
4029 independent reflections
2250 reflections with *I* > 2σ(*I*)
*R*_int_ = 0.032
3 standard reflections every 200 reflections intensity decay: 1%

#### Refinement

*R*[*F* ^2^ > 2σ(*F* ^2^)] = 0.063
*wR*(*F* ^2^) = 0.152
*S* = 1.01
4029 reflections
289 parameters
1 restraint
H-atom parameters constrained
Δρ_max_ = 0.49 e Å^−3^
Δρ_min_ = −0.47 e Å^−3^

### 

Data collection: *CAD-4 EXPRESS* (Enraf–Nonius, 1994 ▶); cell refinement: *CAD-4 EXPRESS*; data reduction: *XCAD4* (Harms & Wocadlo, 1995 ▶); program(s) used to solve structure: *SHELXS97* (Sheldrick, 2008 ▶); program(s) used to refine structure: *SHELXL97* (Sheldrick, 2008 ▶); molecular graphics: *SHELXTL* (Sheldrick, 2008 ▶); software used to prepare material for publication: *PLATON* (Spek, 2009 ▶).

## Supplementary Material

Crystal structure: contains datablock(s) global, I. DOI: 10.1107/S1600536812024154/hb6699sup1.cif

Structure factors: contains datablock(s) I. DOI: 10.1107/S1600536812024154/hb6699Isup2.hkl

Supplementary material file. DOI: 10.1107/S1600536812024154/hb6699Isup3.cml

Additional supplementary materials:  crystallographic information; 3D view; checkCIF report

## Figures and Tables

**Table 1:** Hydrogen-bond geometry (Å, °)

*D*—H⋯*A*	*D*—H	H⋯*A*	*D*⋯*A*	*D*—H⋯*A*
N3—H3*A*⋯O4	0.86	1.95	2.790 (6)	167
C9—H9*A*⋯O3^i^	0.93	2.55	3.271 (7)	135
C10—H10*A*⋯O3^ii^	0.93	2.49	3.263 (7)	140
C10—H10*A*⋯O5^ii^	0.93	2.42	3.340 (7)	168
C19—H19*A*⋯O3	0.93	2.54	3.276 (7)	137

Symmetry codes: (i) 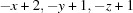; (ii) 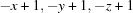.

**Fig. 1.**
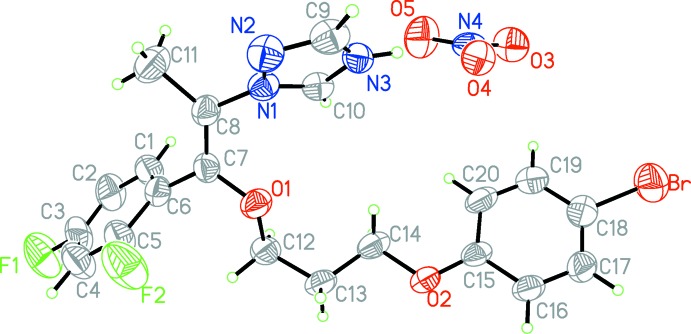
The molecular structure of the title molecule, with displacement ellipsoids drawn at 30% probability levels.

**Fig. 2.**
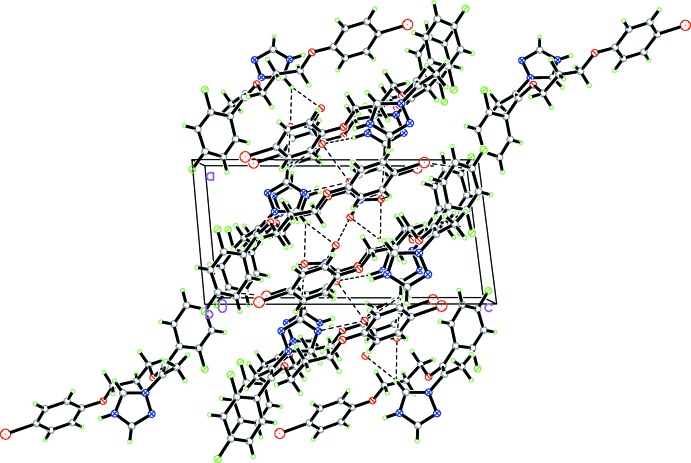
The packing diagram of the title compound. Hydron bonds are shown as dashed lines.

## supplementary crystallographic information

### Comment

Triazole derivatives such as Voriconazole ((*2R,3S*)-2-(2,4-difluorophenyl) -3-(5-fluoropyrimidin-4-yl)-1-(1*H*-1,2,4-triazol-1-yl) butan-2-ol) and Posaconazole (4-(4-(4-(4-(((*3R,5R*)-5-(2,4-difluorophenyl)-5-(1,2,4- triazol-1-ylmethyl)oxolan-3-yl)methoxy)phenyl)piperazin-1-yl)phenyl)- 2-((*2S,3S*)-2-hydroxypentan-3-yl)-1,2,4-triazol-3-one) are safe and effective antifungal agents. (Jeu *et al.*, 2003; Fromtling & Castaner, 1996) As part of our studies on the synthesis of new triazole derivatives, the crystal structure of the title compound was determined.

In the molecular structure of the title compound the double bond has a Z conformation. In the crystal structure the anions and cations are connected via N—H···O and C—H···O hydrogen bonding (Table 1 and Fig. 2).

### Experimental

3 g (0.01 mol) 1-(2,4-difluorophenyl)-2-(1,2,4-triazol)-1-y1)propan-1-one, 10 g of a 50% aqueous sodium hydroxide, 15 ml toluene and 1.5 ml of a 40% aqueous solution of tetrabutyl ammonium hydroxide are mixed and heated to 323.15 K under vigorous stirring. 2.93 g (0.01 mol) 1-bromo-3-(4-bromophenoxy)-propane, dissolved in 10 ml toluene, is instilled into the stirred and warmed solution in the course of 10 h. The mixture is subsequently stirred for another 20 h at 323.15 K. The reaction mixture is mixed with as much water and chloroform so that the aqueous phase becomes lighter than the organic phase. Thereafter, the organic and aqueous phases are separated. The organic phase is dried with sodium sulfate. The solvents are distilled under reduced pressure. The remaining residue is a dark oil that is diluted with 10 ml 2-propanol and then adjusted to a PH-value of 2 by means of 30% aqueous nitric acid. The thus derived nitric acid solution is then cooled in the refrigerator. The impure precipitated product herein is subsequently crystallized from a 1:1 mixture of ethyl acetate and ethanol. The purified product may be analytically identified as an approximately pure *Z*-isomer of propylene nitrate. Colourless plates of the title compound were obtained by slow evaporation of an ethanol solution. Details on the synthesis can be found in the literature reported by Ludwig & Kurt (1985).

### Refinement

H atoms were positioned geometrically with C—H = 0.93 and 0.97 Å for aromatic and methylene H atoms, respectively, and with N—H = 0.86 Å for triazole H atom, and constrained to ride on their parent atoms, with *U*_iso_(H) = 1.2 (or 1.5 for methyl groups) times *U*_eq_(C).

### Crystal data

**Table:**

C_20_H_19_BrF_2_N_3_O_2_^+^·NO_3_^−^	*Z* = 2
*M**_r_* = 513.30	*F*(000) = 520
Triclinic, *P*1	*D*_x_ = 1.552 Mg m^−^^3^
Hall symbol: -P 1	Mo *K*α radiation, λ = 0.71073 Å
*a* = 8.3030 (17) Å	Cell parameters from 25 reflections
*b* = 8.4260 (17) Å	θ = 9–13°
*c* = 16.170 (3) Å	µ = 1.93 mm^−^^1^
α = 91.10 (3)°	*T* = 293 K
β = 95.80 (3)°	Plate, colorless
γ = 102.30 (3)°	0.20 × 0.10 × 0.10 mm
*V* = 1098.7 (4) Å^3^	

### Data collection

**Table:**

Enraf–Nonius CAD-4 diffractometer	2250 reflections with *I* > 2σ(*I*)
Radiation source: fine-focus sealed tube	*R*_int_ = 0.032
Graphite monochromator	θ_max_ = 25.4°, θ_min_ = 1.3°
ω/2θ scans	*h* = 0→10
Absorption correction: ψ scan (North *et al.*, 1968)	*k* = −10→9
*T*_min_ = 0.699, *T*_max_ = 0.831	*l* = −19→19
4329 measured reflections	3 standard reflections every 200 reflections
4029 independent reflections	intensity decay: 1%

### Refinement

**Table:**

Refinement on *F*^2^	Primary atom site location: structure-invariant direct methods
Least-squares matrix: full	Secondary atom site location: difference Fourier map
*R*[*F*^2^ > 2σ(*F*^2^)] = 0.063	Hydrogen site location: inferred from neighbouring sites
*wR*(*F*^2^) = 0.152	H-atom parameters constrained
*S* = 1.01	*w* = 1/[σ^2^(*F*_o_^2^) + (0.075*P*)^2^] where *P* = (*F*_o_^2^ + 2*F*_c_^2^)/3
4029 reflections	(Δ/σ)_max_ = 0.001
289 parameters	Δρ_max_ = 0.49 e Å^−^^3^
1 restraint	Δρ_min_ = −0.47 e Å^−^^3^

### Special details

**Table:**

Geometry. All e.s.d.'s (except the e.s.d. in the dihedral angle between two l.s. planes) are estimated using the full covariance matrix. The cell e.s.d.'s are taken into account individually in the estimation of e.s.d.'s in distances, angles and torsion angles; correlations between e.s.d.'s in cell parameters are only used when they are defined by crystal symmetry. An approximate (isotropic) treatment of cell e.s.d.'s is used for estimating e.s.d.'s involving l.s. planes.
Refinement. Refinement of *F*^2^ against ALL reflections. The weighted *R*-factor *wR* and goodness of fit *S* are based on *F*^2^, conventional *R*-factors *R* are based on *F*, with *F* set to zero for negative *F*^2^. The threshold expression of *F*^2^ > σ(*F*^2^) is used only for calculating *R*-factors(gt) *etc*. and is not relevant to the choice of reflections for refinement. *R*-factors based on *F*^2^ are statistically about twice as large as those based on *F*, and *R*- factors based on ALL data will be even larger.

### Fractional atomic coordinates and isotropic or equivalent isotropic displacement parameters (Å^2^)

**Table:**

	*x*	*y*	*z*	*U*_iso_*/*U*_eq_	
Br	0.98343 (10)	0.93868 (9)	0.81164 (4)	0.0842 (3)	
O1	0.5622 (4)	0.7633 (4)	0.2460 (2)	0.0504 (9)	
N1	0.6426 (5)	0.4746 (5)	0.2587 (2)	0.0415 (10)	
F1	−0.0592 (5)	0.7759 (6)	−0.0124 (3)	0.1075 (14)	
C1	0.1701 (7)	0.5970 (7)	0.1459 (3)	0.0567 (15)	
H1A	0.1495	0.5219	0.1871	0.068*	
O2	0.7624 (4)	1.0648 (4)	0.4584 (2)	0.0546 (10)	
F2	0.5119 (5)	0.8501 (5)	0.0607 (2)	0.0953 (13)	
N2	0.7812 (5)	0.4226 (5)	0.2392 (3)	0.0516 (11)	
C2	0.0372 (7)	0.6312 (8)	0.0959 (4)	0.0686 (17)	
H2B	−0.0714	0.5805	0.1027	0.082*	
N3	0.7821 (5)	0.4579 (5)	0.3707 (3)	0.0445 (10)	
H3A	0.8152	0.4614	0.4230	0.053*	
C3	0.0711 (8)	0.7408 (8)	0.0371 (4)	0.0676 (17)	
C4	0.2289 (8)	0.8143 (8)	0.0214 (4)	0.0734 (18)	
H4A	0.2481	0.8853	−0.0216	0.088*	
C5	0.3555 (7)	0.7772 (7)	0.0724 (3)	0.0605 (15)	
C6	0.3333 (6)	0.6713 (6)	0.1366 (3)	0.0456 (12)	
C7	0.4737 (6)	0.6405 (6)	0.1930 (3)	0.0425 (12)	
C8	0.5168 (6)	0.4976 (6)	0.1943 (3)	0.0449 (12)	
C9	0.8628 (7)	0.4159 (6)	0.3118 (4)	0.0504 (14)	
H9A	0.9642	0.3854	0.3204	0.060*	
C10	0.6445 (6)	0.4938 (6)	0.3394 (3)	0.0402 (12)	
H10A	0.5645	0.5261	0.3681	0.048*	
C11	0.4530 (8)	0.3536 (7)	0.1346 (4)	0.0704 (18)	
H11A	0.3712	0.3784	0.0935	0.106*	
H11B	0.4040	0.2615	0.1645	0.106*	
H11C	0.5432	0.3289	0.1077	0.106*	
C12	0.4855 (7)	0.8956 (6)	0.2671 (3)	0.0530 (14)	
H12A	0.3866	0.8547	0.2941	0.064*	
H12B	0.4542	0.9490	0.2172	0.064*	
C13	0.6100 (7)	1.0130 (6)	0.3248 (3)	0.0530 (14)	
H13A	0.5696	1.1117	0.3321	0.064*	
H13B	0.7131	1.0415	0.2997	0.064*	
C14	0.6429 (7)	0.9442 (6)	0.4086 (3)	0.0528 (14)	
H14A	0.5411	0.9168	0.4348	0.063*	
H14B	0.6851	0.8463	0.4023	0.063*	
C15	0.8075 (6)	1.0270 (6)	0.5371 (3)	0.0446 (12)	
C16	0.9266 (7)	1.1447 (6)	0.5838 (4)	0.0536 (14)	
H16A	0.9712	1.2414	0.5598	0.064*	
C17	0.9793 (7)	1.1207 (7)	0.6646 (4)	0.0565 (15)	
H17A	1.0592	1.2002	0.6952	0.068*	
C18	0.9121 (7)	0.9758 (7)	0.7007 (3)	0.0545 (14)	
C19	0.7945 (7)	0.8595 (7)	0.6547 (4)	0.0562 (15)	
H19A	0.7494	0.7628	0.6786	0.067*	
C20	0.7436 (7)	0.8845 (6)	0.5745 (4)	0.0568 (15)	
H20A	0.6643	0.8041	0.5441	0.068*	
O4	0.8591 (4)	0.5077 (4)	0.5425 (2)	0.0537 (9)	
N4	0.7288 (6)	0.4501 (5)	0.5751 (3)	0.0502 (11)	
O3	0.7345 (5)	0.4623 (5)	0.6516 (3)	0.0658 (11)	
O5	0.6014 (5)	0.3882 (5)	0.5316 (3)	0.0775 (13)	

### Atomic displacement parameters (Å^2^)

**Table:**

	*U*^11^	*U*^22^	*U*^33^	*U*^12^	*U*^13^	*U*^23^
Br	0.0977 (6)	0.0922 (6)	0.0618 (4)	0.0230 (4)	−0.0016 (4)	0.0056 (4)
O1	0.048 (2)	0.042 (2)	0.061 (2)	0.0136 (17)	−0.0076 (18)	−0.0066 (17)
N1	0.039 (2)	0.039 (2)	0.048 (3)	0.0110 (19)	0.005 (2)	−0.0007 (19)
F1	0.083 (3)	0.147 (4)	0.093 (3)	0.038 (3)	−0.022 (2)	0.035 (3)
C1	0.055 (4)	0.059 (4)	0.051 (3)	0.004 (3)	−0.001 (3)	0.010 (3)
O2	0.057 (2)	0.034 (2)	0.065 (2)	0.0005 (17)	−0.0107 (19)	−0.0009 (18)
F2	0.066 (2)	0.120 (3)	0.091 (3)	−0.004 (2)	0.007 (2)	0.046 (2)
N2	0.045 (3)	0.059 (3)	0.054 (3)	0.017 (2)	0.010 (2)	−0.003 (2)
C2	0.046 (4)	0.089 (5)	0.065 (4)	0.012 (3)	−0.013 (3)	0.003 (4)
N3	0.036 (2)	0.041 (2)	0.054 (3)	0.006 (2)	−0.003 (2)	0.004 (2)
C3	0.065 (4)	0.091 (5)	0.049 (3)	0.032 (4)	−0.016 (3)	0.010 (3)
C4	0.077 (5)	0.080 (5)	0.061 (4)	0.014 (4)	−0.004 (4)	0.024 (3)
C5	0.055 (4)	0.070 (4)	0.050 (3)	0.002 (3)	−0.002 (3)	0.014 (3)
C6	0.046 (3)	0.048 (3)	0.040 (3)	0.008 (3)	−0.003 (2)	−0.003 (2)
C7	0.041 (3)	0.046 (3)	0.038 (3)	0.007 (3)	−0.002 (2)	0.002 (2)
C8	0.042 (3)	0.045 (3)	0.045 (3)	0.008 (2)	−0.003 (2)	−0.003 (2)
C9	0.038 (3)	0.052 (3)	0.066 (4)	0.018 (3)	0.013 (3)	−0.003 (3)
C10	0.031 (3)	0.040 (3)	0.051 (3)	0.010 (2)	0.009 (2)	0.003 (2)
C11	0.070 (4)	0.062 (4)	0.075 (4)	0.016 (3)	−0.009 (3)	−0.020 (3)
C12	0.051 (3)	0.044 (3)	0.063 (3)	0.015 (3)	−0.009 (3)	0.002 (3)
C13	0.058 (4)	0.032 (3)	0.065 (4)	0.006 (3)	−0.005 (3)	−0.004 (3)
C14	0.047 (3)	0.036 (3)	0.070 (4)	0.001 (3)	0.002 (3)	−0.005 (3)
C15	0.043 (3)	0.029 (3)	0.062 (3)	0.010 (2)	0.004 (3)	−0.005 (2)
C16	0.050 (3)	0.033 (3)	0.074 (4)	0.006 (3)	0.000 (3)	0.002 (3)
C17	0.051 (3)	0.050 (4)	0.067 (4)	0.014 (3)	−0.006 (3)	−0.004 (3)
C18	0.051 (3)	0.057 (4)	0.058 (3)	0.019 (3)	0.006 (3)	−0.005 (3)
C19	0.063 (4)	0.044 (3)	0.061 (4)	0.009 (3)	0.011 (3)	0.003 (3)
C20	0.054 (4)	0.034 (3)	0.077 (4)	0.000 (3)	0.002 (3)	−0.007 (3)
O4	0.035 (2)	0.057 (2)	0.067 (2)	0.0034 (17)	0.0080 (18)	0.0052 (19)
N4	0.042 (3)	0.038 (3)	0.074 (3)	0.012 (2)	0.012 (3)	0.007 (2)
O3	0.074 (3)	0.071 (3)	0.056 (3)	0.016 (2)	0.020 (2)	0.011 (2)
O5	0.038 (2)	0.091 (3)	0.091 (3)	−0.010 (2)	0.003 (2)	0.000 (3)

### Geometric parameters (Å, º)

**Table:**

Br—C18	1.884 (6)	C9—H9A	0.9300
O1—C7	1.364 (6)	C10—H10A	0.9300
O1—C12	1.448 (6)	C11—H11A	0.9600
N1—C10	1.309 (6)	C11—H11B	0.9600
N1—N2	1.379 (5)	C11—H11C	0.9600
N1—C8	1.445 (6)	C12—C13	1.500 (7)
F1—C3	1.368 (6)	C12—H12A	0.9700
C1—C2	1.386 (7)	C12—H12B	0.9700
C1—C6	1.392 (7)	C13—C14	1.507 (7)
C1—H1A	0.9300	C13—H13A	0.9700
O2—C15	1.352 (6)	C13—H13B	0.9700
O2—C14	1.430 (6)	C14—H14A	0.9700
F2—C5	1.347 (6)	C14—H14B	0.9700
N2—C9	1.304 (6)	C15—C20	1.383 (7)
C2—C3	1.346 (8)	C15—C16	1.391 (7)
C2—H2B	0.9300	C16—C17	1.368 (7)
N3—C10	1.300 (6)	C16—H16A	0.9300
N3—C9	1.303 (6)	C17—C18	1.395 (8)
N3—H3A	0.8600	C17—H17A	0.9300
C3—C4	1.375 (8)	C18—C19	1.374 (7)
C4—C5	1.363 (8)	C19—C20	1.356 (7)
C4—H4A	0.9300	C19—H19A	0.9300
C5—C6	1.382 (7)	C20—H20A	0.9300
C6—C7	1.476 (7)	O4—N4	1.259 (5)
C7—C8	1.327 (7)	N4—O5	1.219 (5)
C8—C11	1.504 (7)	N4—O3	1.235 (5)
			
C7—O1—C12	118.7 (4)	C8—C11—H11C	109.5
C10—N1—N2	110.9 (4)	H11A—C11—H11C	109.5
C10—N1—C8	128.3 (4)	H11B—C11—H11C	109.5
N2—N1—C8	120.8 (4)	O1—C12—C13	107.4 (4)
C2—C1—C6	122.1 (5)	O1—C12—H12A	110.2
C2—C1—H1A	119.0	C13—C12—H12A	110.2
C6—C1—H1A	119.0	O1—C12—H12B	110.2
C15—O2—C14	117.2 (4)	C13—C12—H12B	110.2
C9—N2—N1	103.0 (4)	H12A—C12—H12B	108.5
C3—C2—C1	117.5 (6)	C12—C13—C14	112.4 (4)
C3—C2—H2B	121.3	C12—C13—H13A	109.1
C1—C2—H2B	121.3	C14—C13—H13A	109.1
C10—N3—C9	110.5 (4)	C12—C13—H13B	109.1
C10—N3—H3A	124.8	C14—C13—H13B	109.1
C9—N3—H3A	124.8	H13A—C13—H13B	107.9
C2—C3—F1	118.0 (6)	O2—C14—C13	107.9 (4)
C2—C3—C4	124.0 (6)	O2—C14—H14A	110.1
F1—C3—C4	118.0 (6)	C13—C14—H14A	110.1
C5—C4—C3	116.3 (6)	O2—C14—H14B	110.1
C5—C4—H4A	121.9	C13—C14—H14B	110.1
C3—C4—H4A	121.9	H14A—C14—H14B	108.4
F2—C5—C4	118.2 (5)	O2—C15—C20	125.7 (5)
F2—C5—C6	117.8 (5)	O2—C15—C16	116.1 (5)
C4—C5—C6	124.0 (6)	C20—C15—C16	118.1 (5)
C5—C6—C1	116.0 (5)	C17—C16—C15	121.1 (5)
C5—C6—C7	122.3 (5)	C17—C16—H16A	119.4
C1—C6—C7	121.7 (5)	C15—C16—H16A	119.4
C8—C7—O1	118.6 (4)	C16—C17—C18	119.4 (5)
C8—C7—C6	122.5 (5)	C16—C17—H17A	120.3
O1—C7—C6	118.8 (4)	C18—C17—H17A	120.3
C7—C8—N1	117.9 (4)	C19—C18—C17	119.6 (5)
C7—C8—C11	127.8 (5)	C19—C18—Br	120.1 (4)
N1—C8—C11	114.4 (4)	C17—C18—Br	120.3 (4)
N3—C9—N2	110.4 (5)	C20—C19—C18	120.5 (5)
N3—C9—H9A	124.8	C20—C19—H19A	119.7
N2—C9—H9A	124.8	C18—C19—H19A	119.7
N3—C10—N1	105.2 (4)	C19—C20—C15	121.3 (5)
N3—C10—H10A	127.4	C19—C20—H20A	119.4
N1—C10—H10A	127.4	C15—C20—H20A	119.4
C8—C11—H11A	109.5	O5—N4—O3	121.9 (5)
C8—C11—H11B	109.5	O5—N4—O4	120.4 (5)
H11A—C11—H11B	109.5	O3—N4—O4	117.7 (5)
			
C10—N1—N2—C9	−1.1 (5)	C10—N1—C8—C7	55.1 (7)
C8—N1—N2—C9	−179.7 (4)	N2—N1—C8—C7	−126.5 (5)
C6—C1—C2—C3	−0.2 (9)	C10—N1—C8—C11	−126.2 (5)
C1—C2—C3—F1	−179.8 (5)	N2—N1—C8—C11	52.2 (6)
C1—C2—C3—C4	3.1 (10)	C10—N3—C9—N2	0.0 (6)
C2—C3—C4—C5	−3.4 (10)	N1—N2—C9—N3	0.6 (6)
F1—C3—C4—C5	179.6 (6)	C9—N3—C10—N1	−0.7 (5)
C3—C4—C5—F2	−178.2 (6)	N2—N1—C10—N3	1.1 (5)
C3—C4—C5—C6	0.6 (10)	C8—N1—C10—N3	179.6 (4)
F2—C5—C6—C1	−179.2 (5)	C7—O1—C12—C13	−179.3 (4)
C4—C5—C6—C1	2.0 (9)	O1—C12—C13—C14	−68.8 (6)
F2—C5—C6—C7	1.8 (8)	C15—O2—C14—C13	179.2 (4)
C4—C5—C6—C7	−177.0 (6)	C12—C13—C14—O2	180.0 (4)
C2—C1—C6—C5	−2.2 (8)	C14—O2—C15—C20	−2.6 (7)
C2—C1—C6—C7	176.7 (5)	C14—O2—C15—C16	178.6 (4)
C12—O1—C7—C8	−158.1 (5)	O2—C15—C16—C17	178.8 (5)
C12—O1—C7—C6	21.7 (6)	C20—C15—C16—C17	−0.1 (8)
C5—C6—C7—C8	−113.0 (6)	C15—C16—C17—C18	−0.2 (8)
C1—C6—C7—C8	68.1 (7)	C16—C17—C18—C19	0.2 (8)
C5—C6—C7—O1	67.2 (7)	C16—C17—C18—Br	179.5 (4)
C1—C6—C7—O1	−111.7 (6)	C17—C18—C19—C20	0.1 (8)
O1—C7—C8—N1	7.3 (7)	Br—C18—C19—C20	−179.3 (4)
C6—C7—C8—N1	−172.5 (4)	C18—C19—C20—C15	−0.3 (9)
O1—C7—C8—C11	−171.2 (5)	O2—C15—C20—C19	−178.5 (5)
C6—C7—C8—C11	9.0 (9)	C16—C15—C20—C19	0.3 (8)

### Hydrogen-bond geometry (Å, º)

**Table:**

*D*—H···*A*	*D*—H	H···*A*	*D*···*A*	*D*—H···*A*
N3—H3*A*···O4	0.86	1.95	2.790 (6)	167
C9—H9*A*···O3^i^	0.93	2.55	3.271 (7)	135
C10—H10*A*···O3^ii^	0.93	2.49	3.263 (7)	140
C10—H10*A*···O5^ii^	0.93	2.42	3.340 (7)	168
C19—H19*A*···O3	0.93	2.54	3.276 (7)	137

Symmetry codes: (i) −*x*+2, −*y*+1, −*z*+1; (ii) −*x*+1, −*y*+1, −*z*+1.
